# Morphometric analysis of paired fibula and mandible for optimal fibular mandibular reconstruction in a Chinese population

**DOI:** 10.1038/s41598-022-26033-0

**Published:** 2022-12-12

**Authors:** Xiaojie Ma, Zhuo Wang, Jinbo Wan, Jiamin Xu, Haoran Wang, Yifeng Bian, Linzhong Wan, Yifei Du, Yuli Wang, Hua Yuan

**Affiliations:** 1 Jiangsu Key Laboratory of Oral Diseases, Nanjing Medical University, Number 1, Shanghai Road, Nanjing, People’s Republic of China 210029; 2 Department of Oral and Maxillofacial Surgery, Affiliated Hospital of Stomatology, Nanjing Medical University, Nanjing, People’s Republic of China 210029

**Keywords:** Anatomy, Health care

## Abstract

To analyze the morphology of paired fibula and mandible aiming to choose optimal fibular segments for mandibular reconstruction in a Chinses population. A total of 118 cases of paired mandible and fibula was collected. All patients were received preoperative cone beam CT (CBCT) scans for mandibular evaluation and CT-angiographical (CTA) examination of the bilateral lower legs, respectively. The cross-sectional morphological differences between proximal (Side P), middle (Side M) and distal (Side D) segments of fibula and anterior, premolar and molar areas of mandible were compared. The most frequent cross-sectional shape at Side D, Side M and Side P portion of fibula was circular (75.4%), triangular (67.8%) and circular (49.2%), respectively. In anterior, premolar and molar areas of mandible, the most of the cross-section was s-shape (90.82%), straight (83.64%) and oblique (91.89%), respectively. The height and width of upper one third (W1) at Side M were significantly larger than those of Side D and Side P (p < 0.0001). There was significantly difference of width of lower one third (W2) among three groups (p < 0.0001). As for the height and widths of mandible, there was significant difference among anterior, premolar and molar regions (p < 0.0001). The rate of height between Side M of fibula and mandible (H (Side M/area)) was significantly larger than H (Side D/area) and H (Side P/area) (p < 0.01). The ratio of W1 between Side D of fibula and mandible (W1 (Side D/area)) was significantly larger than that of W1 (Side M/area) and W1 (side P/area) (p < 0.05). As for the ratio of W2 between fibula and mandible (W2 (plane/area)), there was significant difference among groups (p < 0.01). The distal and middle segments of fibula were suitable for reconstructing the anterior area of mandible and the proximal segment of fibula was more compatible with the premolar and molar areas of mandible.

**Clinical Relevance** Presurgical morphometric analysis of paired fibula and mandible aids for optimal fibular-based mandibular reconstruction.

## Introduction

Considering the advantages of being able to repair bone and soft tissues defect, the free fibular flap has become the workhorse flap for segmental mandibular reconstruction^1,2^. In the past decades, significant progress has been made in fibular mandibular reconstruction, from conventional free-hand technique to preoperative virtual surgical planning, rapid prototyping and three-dimensional (3D) printing cutting jigs, prefabricated titanium plates and navigation guidance^3^. All these advancements improve precise contouring and positioning of fibula free flaps to achieve a symmetrical facial appearance^4^. Nevertheless, fibula-based mandibular reconstruction remains a major challenge in regard of functional occlusive rehabilitation.

The height of reconstructed neomandible is significantly lower than that of the original bone, which makes implant-retained prosthetic rehabilitation difficult. It was reported that only 4.2% and 21.1% of the harvestable bone length could be suitable for inserting dental implants in females and males, respectively^5^. Also, as shown in a CT-based study, the cross-sectional morphology of transferred fibulae could be classified into two types including apex and nonapex, and the ridge at the apex was too narrow for dental implants^6^. However, the shape of fibula varies along its length and some regions of fibula could meet the height and width for dental implants^7^. Therefore, it is better to evaluate the morphological features of fibula and mandible preoperatively, for the sake of choosing optimal fibular segments for mandibular reconstruction and subsequently dental implantation.

In the present study, we performed a morphometric analysis of fibula and mandible in the same individual who underwent fibula-based reconstruction. The morphological differences between proximal, middle and distal segments of fibula and anterior, premolar and molar areas of mandible were compared with the purpose of selecting the optimal fibular region for mandibular reconstruction. To the best of our knowledge, this was the first comparative study based on paired fibula-mandible in a Chinese population.

## Patients and methods

### Ethics statements

The study was approved by the Ethics Committee of the School of Stomatology, Nanjing Medical University, China (PJ2020-119-001). The study was performed in accordance with the Declaration of Helsinki, and written informed consent was obtained from all patients to publish their clinical information and digital images.

### Patients and data collection

From March 2015 to December 2021, a total of 118 consecutive patients who undergoing free fibula flaps for mandible or maxillary reconstructions at the Department of Oral and Maxillofacial Surgery, Stomatological Hospital affiliated to Nanjing Medical University was retrospectively reviewed. All cases were clinically examined to confirm the primary lesions. Exclusion criteria were as follows: patients were less than 18 years old; patients with bone metabolic disease, such as hyperparathyroidism and diabetic mellitus. Due to the availability of mandibular data, this study also included 4 cases of fibula-based maxillary reconstruction.

All patients were received preoperative CBCT (NewTom VGi, Verona, Italy) scans for mandibular evaluation and CT-angiographical (CTA) examination of the bilateral lower legs, respectively. The CBCT images were acquired at 80 kV and 10 mA, with scanning layer thickness of 0.25 mm. The CTA acquisition parameters were 120 kV and 150 mA, with a tube rotation speed of 0.3 s, in a 256 slice multi-slice computed tomography (MSCT) by Brilliance iCT (Philips Healthcare, Netherlands) in the Department of Radiology, Chinese Traditional Hospital of Jiangsu Province. The digital imaging and communication in medicine (DICOM) data of CBCT and CTA was imported into a three-dimensional reconstruction software Proplan (Proplan, Materialise, Belgium).

### Definition of landmarks

**Figure 1:**
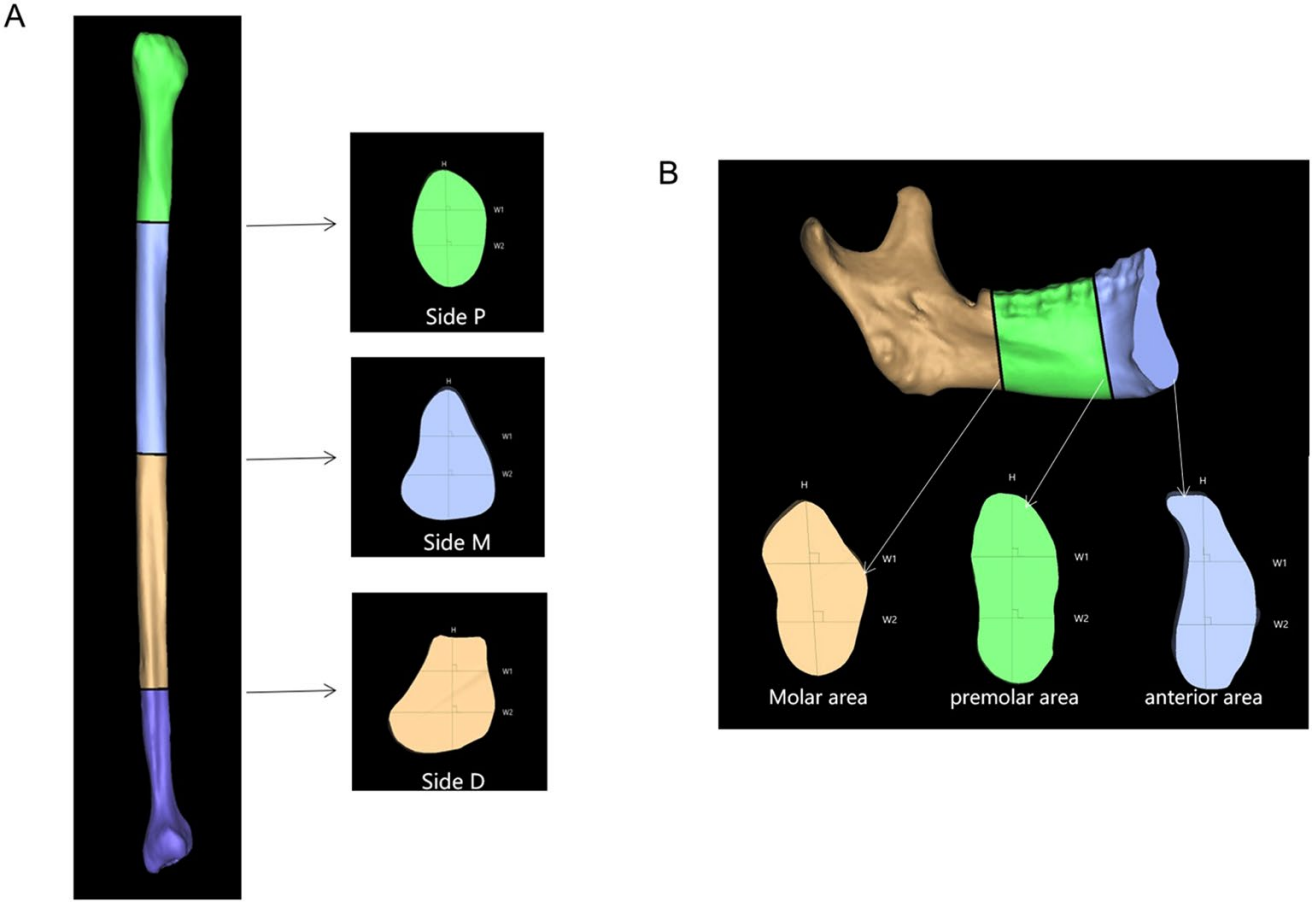
Selected cross-sectional shapes and landmarks of height and widths in paired fibula (**A**) and mandible (**B**).

The sections of mandible and fibula of each patient were reviewed to create a three-dimensional model based on DICOM data of CBCT and CTA, respectively. Fibula morphology was evaluated at three cross-sectional segments with landmarks as follows: Side D was set as 80 mm from the distal end of fibula, Side M was the midpoint of fibula, and Side P was 80 mm from the proximal side of fibula (Fig. 1A). In terms of mandibular morphology, the landmarks were set as transverse plane of anterior, premolar and molar areas. The anterior and premolar areas were located at the incisors and the premolars, respectively. The molar area was located at the distal of the second molar (Fig. 1B)^8^.

Each cross-sectional slide was calculated including the vertical height and transverse widths according to previous studies^8,9^. The vertical height was determined and measured by a line drawn through the long axis of the fibula or mandibular cross-sectional area, with the longest distance, which was marked as H. The transverse widths measurements were recorded perpendicular to the long axis line at two locations, one of which was drawn at upper one third of vertical height marked as W1, and the other was at lower one third of the vertical height marked as W2 (Fig. 1). The vertical height and transverse widths of the selected sections from each mandible and fibula was measured 3 times by two independent observers (Xiaojie Ma and Zhuo Wang) using Proplan software.

### Protocol for the analysis of measurements

In order to investigate optimal fibula segments fibula suitable for mandibular reconstruction, we compare the height and widths at the selected planes of fibula and mandible in each patient. The ratio of height was calculated as the height at Side D, Side M and Side P of fibula/the height at anterior, premolar and molar areas of mandible, expressed as H (plane/area). Similarly, the ratio of width was calculated as the W1 or W2 at Side D, Side M and Side P of fibula/ the W1 or W2 at anterior, premolar and molar areas of mandible, expressed as W1 (plane/area) and W2 (plane /area). The closer the value is to 1, the higher the morphological similarity is.

### Statistical analysis

Data are expressed as the mean ± SD (standard deviation, SD) and statistically analyzed with SPSS 23.0 software (SPSS Inc, IL). The difference among three groups was evaluated using a one-way analysis of variance with a Tukey post hoc test and the two groups were compared using a Wilcoxon matched pairs test. Statistical significance was set at p < 0.05.

### Informed consent

Written informed consent was obtained from all patients for using the CBCT scans for mandibular evaluation and CT-angiographical (CTA) examination of the bilateral lower legs.

## Results

### Population characteristics

The demographic and clinical data of the enrolled patients were given in Table 1. Half of the cases was related to a diagnosis of malignancy, with squamous cell carcinoma being the pathologic diagnosis of a highest frequency (n = 59, 50%). The secondary indication was benign neoplastic lesions (n = 44, 39.27%), most of which was ameloblastoma. The rest of the cases were caused by infection or osteoradionecrosis. Patient characteristics were obtained from the medical record, including 78 males and 40 females, with an average age of 50.65 years (range, 21–77 years).

**Table 1:** The demographic and clinical data of the enrolled patients.

Variable	N (%)
Number	118
Mean age ± SD, year	50.65 ± 14.29
**Sex**	
Male	78 (66.10%)
Female	40 (33.90%)
**Diagnosis**	
SCC	59 (50.00%)
Other malignancies	12 (10.17%)
Benign lesions	44 (37.29%)
Infection	1 (0.85%)
Osteoradionecrosis	2 (1.69%)
**Lesion location**	
Mandible	104 (96.61%)
Maxillary	4 (3.39%)

### Morphometric analysis of fibula and mandible

We analyzed the morphology at the planes of Side D, M and P based on 3D reconstruction of fibula and found 3 types including circular, triangular and square according to the classification of a previous study (Fig. 1A)^8^. Statistically, a total of 87 cases of the cross-section was circular (75.4%), 18 (15.3%) was triangular and 11 (9.3%) was square at Side D. At Side M, triangular was observed in 80 cases (67.8%), square was in 36 cases (30.5%), circular was in 2 cases (1.7%). At Side P, 58 cases (49.2%) were triangular, 48 cases (40.7%) were square and 12 cases (10.1%) were circular (Table 2).

**Table 2:** The percentage of morphology of fibula and mandible at the selected cross-sections.

Fibula	Circular (%)	Triangular (%)	Square (%)	Total
Side P	89 (75.4%)	18 (15.3%)	11 (9.3%)	118
Side M	2 (1.7%)	80 (67.8%)	36 (30.5%)	118
Side D	12 (10.1%)	58 (49.2%)	48 (40.7%)	118

Mandible	S-shape (%)	Straight (%)	Oblique (%)	Total
Anterior area	89 (90.82%)	8 (8.16%)	1 (1.02%)	98
Premolar area	6 (5.45%)	92 (83.64%)	12 (10.91%)	110
Molar area	0	9 (8.11%)	102 (91.89%)	111

Six cases were excluded since the primary lesions involved nearly total mandible could not be calculated. Besides, 10 cases of anterior area, 2 cases of premolar area and 1 cases of molar area were excluded as these regions were related to primary lesion or performed surgeries before. According to our results of the selected areas, we found 3 types of morphology: straight, oblique, and s-shape (Fig. 1B). In anterior area, the most of the cross-section was s-shape (n = 89, 90.82%), while 8 Sections (8.16%) were straight and 1 (1.02%) was oblique. In premolar area, 92 Sections (83.64%) were straight, 12 Sections (10.91%) were oblique and 6 Sections (5.45%) were s-shape. In molar area, 102 Sections (91.89%) were oblique and 9 Sections (8.11%) were straight (Table 2).

The height and widths of fibula/mandible at Side P, Side M and Side D were shown in Tables 3 and 4, respectively. The height of fibula at Side M was15.61 ± 1.77 mm, significantly larger than14.67 ± 1.58 mm of Side D and 14.45 ± 1.57 of Side P (p < 0.0001), while the multiple comparison demonstrated no significant difference between Side P and Side D (p = 0.29). The W1 at Side M was 9.56 ± 1.35 mm, which was significantly larger than 8.32 ± 1.64 mm of Side M and 8.31 ± 1.55 mm of Side D (p < 0.0001), but no difference was found between Side M and Side P (p = 0.95). In terms of W2, there was significantly difference among three groups (p < 0.0001). As for the height and widths of mandible, there was significant difference among anterior, premolar and molar regions (p < 0.0001). Interestingly, the multiple comparison showed a significant difference between heights of anterior and premolar areas (p = 0.04, 95% CI 0.04–1.67).

**Table 3:** The height and width of each section of the fibula.

Variable	Side P (n = 118)	Side M (n = 118)	Side D (n = 118)	Overall	Multiple comparison					
					P-M		P-D		M-D	
					95% CI	P	95% CI	P	95% CI	P
Height	14.67 ± 1.58	15.61 ± 1.77	14.45 ± 1.57	< 0.0001	− 1.35 to − 0.51	< 0.0001	− 0.19 to 0.65	0.29	0.73 to 1.57	< 0.0001
Width 1	9.56 ± 1.35	8.32 ± 1.64	8.31 ± 1.55	< 0.0001	0.85 to 1.62	< 0.0001	0.86 to 1.63	< 0.0001	− 0.38 to 0.40	0.95
Width 2	8.84 ± 0.89	10.79 ± 1.27	12.62 ± 1.46	< 0.0001	− 3.0 to − 2.45	< 0.0001	− 2.00 to − 1.39	< 0.0001	0.74 to 1.38	< 0.0001

**Table 4:** The height and width of each section of the mandible.

Variable	Anterior area (n = 98)	Premolar area (n = 110)	Molar area (n = 111)	Overall	Multiple comparison					
					Anterior-Premolar		Anterior-Molar		Premolar-Molar	
					95% CI	P	95% CI	P	95% CI	P
Height	31.11 ± 3.41	30.25 ± 2.89	26.76 ± 2.63	< 0.0001	0.04 to 1.67	0.04	3.53 to 5.15	< 0.0001	2.70 to 4.28	< 0.0001
Width 1	8.71 ± 1.84	11.58 ± 1.62	15.35 ± 1.69	< 0.0001	− 3.34 to − 2.41	< 0.0001	− 7.11 to − 6.17	< 0.0001	− 4.22 to − 3.31	< 0.0001
Width 2	13.42 ± 1.56	10.79 ± 1.27	12.62 ± 1.46	< 0.0001	2.00 to 2.78	< 0.0001	0.41 to 1.19	< 0.0001	− 1.97 to − 1.21	< 0.0001

### Optimal selection of fibula for mandibular reconstruction

**Figure 2:**
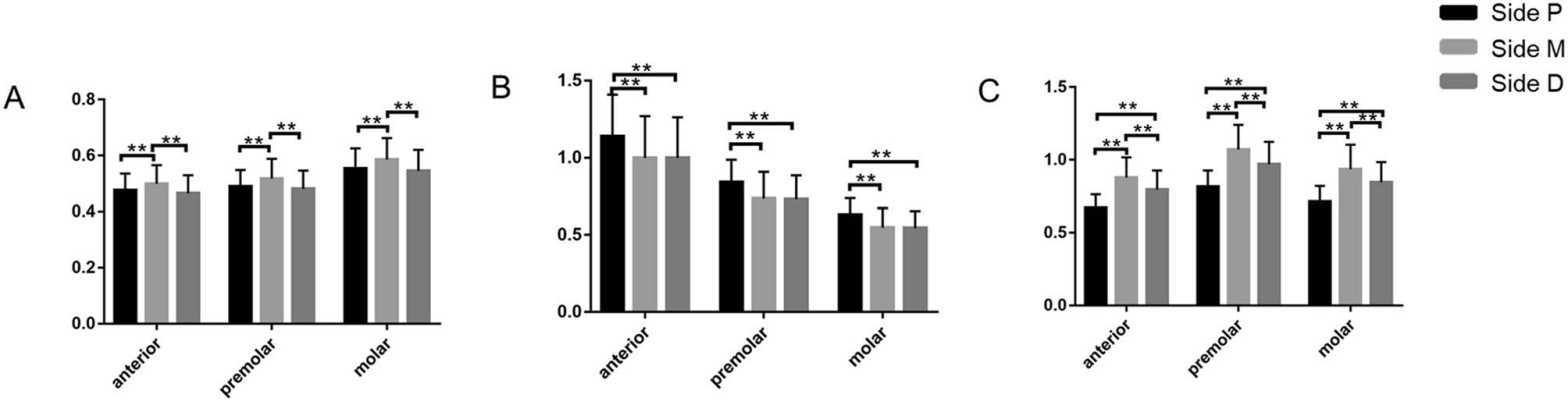
The ratio of height (widths) of each section of fibula to height (width) of different areas of mandible: (**A**) The ratio of height. (**B**) The ratio of W1. (**C**) The ratio of W2.

As shown in Fig. 2A, no matter what areas concerned, the data of H (Side M/area) was significantly larger than H (Side D/area) and H (Side P/area) (p < 0.01). Furthermore, H (Side M/anterior), H (Side M/premolar), H (Side P/molar), H (Side M/molar) and H ((Side D/molar) was 0.50 ± 0.07, 0.52 ± 0.07, 0.55 ± 0.07, 0.59 ± 0.08 and 0.55 ± 0.08, respectively. The rest of data was less than 0.5 (Supplementary Table 1).

Similarly, the data of W1 (Side D/area) was significantly larger than that of W1(Side M/area) and W1(side P/area) (p < 0.01) (Fig. 2B). Specifically, W1 (Side P/anterior area), W1 (Side M/anterior area) and W1 (Side D/anterior area) were 1.14 ± 0.27, 1.00 ± 0.27 and 1.00 ± 0.26, respectively, all of which were more than 1. Besides, W1 (Side P / premolar), W1 (Side M/ premolar) and W1 (Side P /premolar) was 0.84 ± 0.15, 0.73 ± 0.17 and 0.73 ± 0.15, respectively, which were closed to 1 (Supplementary Table 1).

As for W2 (plane/area), there was significant difference among W2 (Side M/area), W2 (Side P/area) and W2 (Side D/area) (p < 0.01) (Fig. 2C). W2 (Side M/premolar) was 1.07 ± 0.17, which was more than 1. Except for W2 (Side P/anterior) which was 0.67 ± 0.09, all others were closed to 1 (Supplementary Table 1).

## Discussion

Modern, high-quality reconstruction of segmental mandibular reconstruction is to achieve symmetrical contour of mandible and provide adequate bone volume for implant-retained prosthetic rehabilitation. Although many issues need to be considered when designing fibula free flaps for mandibular reconstruction, such as defect size, number of fibula segments and perforators of skin island as well as pedicle orientation and length, anatomical studies concerning mandibular or fibular morphology were performed to achieve aesthetic and functional outcomes^5–8^. Our morphometric study differed from previous reports by the methodology used. From clinical observations, one person’s mandible and fibula show the same size trend; however, few studies compared the morphology of fibulae and mandible in the same individuals, which was the major advantage of the present study, consisting of a total of 118 paired fibula and mandible in a Chinese population. Also, the similarity of contour in paired fibula and mandible was analyzed in order to choose the optimal segment of fibula for mandibular reconstruction.

Appropriate assessments of graft morphology and optimal bone contour restoration are required to improve symmetry of the lower facial profile, which could be discriminated within the range of millimeters^9,10^. A recent study compared contour restoration of mandibular body defects with “Single shot” and osteotomized fibula flaps (SS-FF and O-FF), iliac crest flaps (ICF) and scapular tip flaps (STF), and demonstrated that O-FF performed best in contour conformance with native mandible^11^. The cross-sectional shape of the fibula at different sites was usually discussed in previous studies. According to the classification of Fujisawa et al.^12^, the cross-sectional shape of fibula on CT images could be divided into 4 types: knife-edged, triangular, circular, and square. Due to different sites concerned, the shapes of the cross-sections at middle of fibula were categorized into three types: triangular, quadrilateral, and irregular^13,14^. In addition to the shape of mandible, German et al.^15^ defined posterior mandibular ridge morphology as 5 types: straight, oblique, s-shape, hourglass, and basal bone. The main purpose of these reports was to determine the possibility of inserting dental implants. As a matter of fact, it could be better to restore the mandible defect in accordance with its original morphology. As shown in Table 2, by virtue of the cross-sectional shapes, 90.82% of mandibular anterior area presented S-shape, and could be best reconstructed with Side M of fibula, 67.8% of which showed triangular shape. The secondary option was Side D of fibula, with 49.2% of triangular. If the mandibular defect occurred in premolar area, 83.64% of which was straight, the best choice was Side P of fibula with 75.4% of circular, or Side D of fibula with 40.7% of square. Mandibular defect of molar area with 91.89% of oblique, could be preferred by Side P of fibula with 75.4% of circular, or Side D of fibula with 40.7% of square. These results indicated that the similarity in cross-sectional shape between fibula and mandible should be evaluated preoperatively, which could provide additional information in fibula harvesting.

Fibula could provide adequate length but limited height or bone thickness in comparison with ilium^5,16^. Since the vertical height of fibula was shorter than that of native mandible, it was recommended to place transferred fibula on the lower edge of mandible to improve cosmesis, or on the upper edge of mandible to reduce the implant height. Thus, the height deficiency of free fibular flap renders the dental implant insertion into reconstructed mandibles to be one of the most challenging procedures for occlusal rehabilitation. According to our results, the height of fibula at Side M was significantly larger than that of Side D and Side P (Table 3). Moreover, the H (Side M/area) was significantly larger than that of H (Side D/area) and H (Side P/area) (Fig. 2A). All these results proved that the middle of the fibula was the optimal segment for mandibular reconstruction in term of bony height. Even so, it should be recognized that the height of the middle of the fibula was only half of the height of the naive mandible, which could be solved in following ways. Double-barrel technique of the fibula in the primary surgery or vertical osteodistraction of the integrated fibula flap in the secondary surgery was also reliable but drove the surgeons to a complicated surgery scenario^17,18^. Sun et.al proposed a convenient free bone grafting method using nonvascularized fibula as an inlay graft to the lower border of the neomandible^19^. Recently, a novel reconstructive customized plate was projected to support the fibular flap at an alveolar bone position above the typical inferior mandibular border^20^. This reconstructive proposal appears to be a valid alternative to restore the vertical height of the reconstructed mandible, and it could be better if the customized plate was combined with optimal fibula segments.

In addition to height, it was evidenced that fibula showed variable bone dimensions and some sites presented inadequate width for dental implants^5^. A digital imaging study suggested that the width of the central section of fibula was the largest compared with that of lateral malleolus and head of the fibula. Moreover, with respect to the available length with adequate bone volume for dental implants, the length near the lateral malleolus was larger than that near the head of fibula^14^. However, our data suggested the maximum of W1 was located at Side P while the maximum of W2 was located at Side D, not at the midpoint of fibula (Table 3). The discrepancy between our and previous results may attribute to inconsistent of cross-sectional regions and different measuring methods. Nevertheless, in regard to fibular width, Side D, Side M and Side P could provide essential bone volume for dental implants of regular diameters of 3.5–4.5 mm. Considering the width of mandible, as shown in Table 4, the optimal segments of fibula for mandibular reconstruction could be variable. The ratio analysis indicated that Side D and Side M of fibula could be favorable for mandibular defect of anterior area, while Side P of fibula was compatible with mandibular defect of premolar and molar areas (Supplementary Table 1). Notably, even in the same position, the maximum and minimum values of ratio analysis often differ by more than three times (for example, W1 ratio of Side M/Anterior ranged from 0.49 to 1.91; W2 ratio of Side D/Anterior ranged from 0.39 to 1.29). These results indicated that the personalized design of mandibular reconstruction based on fibular measurements is particularly necessary.

The primary issues of fibular based mandible reconstruction are the pedicle orientation and length as well as numbers of segments. However, to some cases with limited mandibular defect, choosing the best-fit couple of fibula segments to repair the mandibular portion could be the first consideration. Based on our results, proper selection of fibular regions could be essential to mandibular reconstructions, especially when the mandibular defect was confined to semi mandible. By morphometric analysis preoperatively, selecting fibula segments with similar contour to the resected mandible could restore the original mandibular contour of the patient to the greatest extent. Also, it could be better to placing fibula segments with adequate bone height in the canine and molar areas could felicitate the implant-supported dental rehabilitation. Both could achieve a favorable aesthetic and functional outcomes of fibular based mandible reconstruction. However, this study did have several shortcomings. First of all, as a retrospective and radiographic investigation, the results could not be verified by clinical outcomes, which is needed in further studies. Moreover, the digital data of mandible and fibula came from CBCT and MSCT, respectively. Although both of them was processed by the same software, the differences of scanning principle and imaging resolution may cause bias to the results. Additionally, we recognized that in most cases of segmental mandible defect, the part of mandibular reconstruction may involve multiple parts of anterior, premolar and molar areas. For such cases with expanded segmental mandible defect, other factors should be prior considered, for example the location and required length of the flap pedicle. Therefore, within the cases of limited mandibular defect, fibula-based mandible reconstruction with optimal fibular segments is reliable.

## Conclusion

This study focused on morphometric analysis of paired fibula and mandible including morphological similarity and measurements of heigh and width in a Chinese population. Within the limitations of this study, we speculated that the distal site of fibula was favorable for mandibular defect at premolar area, the middle of fibula for anterior area, and the proximal of fibula for molar area. In regard of height and width, the distal and middle sites of fibula were suitable for the anterior area of the mandible and the proximal segments of fibula was more compatible with the premolar and molar areas of mandible.

## Supplementary Information

Supplementary Information.

Supplementary Table 1.

## Data Availability

All data generated or analyzed during this study are included in this published article [See the supplementary file with the name of Calculation of all cases].
