# Supplementary material for: Morphometric analysis of paired fibula and mandible for optimal fibular mandibular reconstruction in a Chinese population
Source: Sci Rep. 2022 Dec 12;12:21460. doi: 10.1038/s41598-022-26033-0 (PMC9744824; doi:10.1038/s41598-022-26033-0)
Supplement: Supplementary file 2 — Supplementary Table 1. [file 41598_2022_26033_MOESM2_ESM.docx]

**Morphometric analysis of paired fibula and mandible for optimal fibular mandibular reconstruction in a Chinese population**

Xiaojie Ma^1,2^, Zhuo Wang^1,2^, Jinbo Wan^1,2^, Jiamin Xu^1,2^, Haoran Wang^1,2^, Yifeng Bian^1,2^, Linzhong Wan^1,2^, Yifei Du^1,2*^, Yuli Wang ^1,2*^, Hua Yuan^1,2*^

Xiaojie Ma, Zhuo Wang and Jinbo Wan contributed equally to this article.

**Authors affiliations:**

a Jiangsu Key Laboratory of Oral Diseases, Nanjing Medical University; Nanjing, People’s Republic of China, 210029;

b Department of Oral and Maxillofacial Surgery, Affiliated Hospital of Stomatology, Nanjing Medical University, Nanjing, People’s Republic of China, 210029;

**Corresponding author**:

YiFei Du. Email: dyf@njmu.edu.cn

Yuli Wang. Email: njykdwyl@njmu.edu.cn

Hua Yuan. Email: 56422269@qq.com

Address: Number 1, Shanghai Road, Nanjing, People’s Republic of China, 210029

Tel: 0086-25-85031875

Fax: 0086-25-86516414

| Variable | Height  (n=98) | | | Width 1  (n=110) | | | Width 2  (n=111) | | |
| --- | --- | --- | --- | --- | --- | --- | --- | --- | --- |
|  | Mean | Range | SD | Mean | Range | SD | Mean | Range | SD |
| Side P/Anterior | 0.48 | 0.35-0.65 | 0.06 | 1.14 | 0.62-1.98 | 0.27 | 0.67 | 0.50-1.05 | 0.09 |
| Side M/Anterior | 0.50 | 0.25-0.68 | 0.07 | 1.00 | 0.49-1.91 | 0.27 | 0.88 | 0.43-1.43 | 0.14 |
| Side D/Anterior | 0.46 | 0.31-0.64 | 0.06 | 1.00 | 0.62-2.06 | 0.26 | 0.80 | 0.39-1.29 | 0.13 |
| Side P/ Premolar | 0.49 | 0.36-0.64 | 0.06 | 0.84 | 0.52-1.23 | 0.15 | 0.82 | 0.61-1.14 | 0.11 |
| Side M/Premolar | 0.52 | 0.25-0.75 | 0.07 | 0.73 | 0.41-1.35 | 0.17 | 1.07 | 0.55-1.64 | 0.17 |
| Side D/Premolar | 0.48 | 0.32-0.71 | 0.07 | 0.73 | 0.50-1.27 | 0.15 | 0.97 | 0.54-1.30 | 0.15 |
| Side P/Molar | 0.55 | 0.38-0.75 | 0.07 | 0.63 | 0.36-0.93 | 0.10 | 0.71 | 0.52-1.10 | 0.10 |
| Side M/Molar | 0.59 | 0.29-0.79 | 0.08 | 0.55 | 0.30-1.06 | 0.13 | 0.94 | 0.48-1.64 | 0.10 |
| Side D/Molar | 0.55 | 0.36-0.76 | 0.08 | 0.54 | 0.36-0.90 | 0.11 | 0.85 | 0.47-1.26 | 0.10 |

Supplementary Table 1 The ratio of height, width 1 and width 2 of fibula to that of mandible at different areas
